# Resveratrol and Endothelial Nitric Oxide

**DOI:** 10.3390/molecules191016102

**Published:** 2014-10-09

**Authors:** Ning Xia, Ulrich Förstermann, Huige Li

**Affiliations:** Department of Pharmacology, Johannes Gutenberg University Medical Center, Obere Zahlbacher Str. 67, Mainz 55131, Germany; E-Mails: xianing@uni-mainz.de (N.X.); ulrich.forstermann@uni-mainz.de (U.F.)

**Keywords:** resveratrol, nitric oxide, eNOS, SIRT1, Nrf2

## Abstract

Nitric oxide (NO) derived from the endothelial NO synthase (eNOS) has antihypertensive, antithrombotic, anti-atherosclerotic and antiobesogenic properties. Resveratrol is a polyphenol phytoalexin with multiple cardiovascular and metabolic effects. Part of the beneficial effects of resveratrol are mediated by eNOS. Resveratrol stimulates NO production from eNOS by a number of mechanisms, including upregulation of eNOS expression, stimulation of eNOS enzymatic activity and reversal of eNOS uncoupling. In addition, by reducing oxidative stress, resveratrol prevents oxidative NO inactivation by superoxide thereby enhancing NO bioavailability. Molecular pathways underlying these effects of resveratrol involve SIRT1, AMPK, Nrf2 and estrogen receptors.

## 1. Endothelial Nitric Oxide

Nitric oxide (NO) is produced by three isoforms of NO synthase (NOS): the neuronal nNOS (NOS I), the inducible iNOS (NOS II) and the endothelial eNOS (NOS III). All NOS isoforms utilize l-arginine and molecular oxygen as the substrates and reduced nicotinamide-adenine-dinucleotide phosphate (NADPH) as co-substrates. Flavin adenine dinucleotide (FAD), flavin mononucleotide (FMN), and (6*R*-)5,6,7,8-tetrahydro-l-biopterin (BH_4_) are cofactors of all NOS isozymes [1].

Under physiological conditions, vascular NO is mainly produced by eNOS. This enzyme is constitutively expressed in the endothelium and is activated by shear stress or by agonists such as bradykinin and acetylcholine. Molecular mechanisms underlying eNOS activation includes elevation of intracellular Ca^2+^ concentration, post-translational modification of the eNOS enzyme (e.g., phosphorylation and acetylation) and protein-protein interaction [2].

Endothelial NO relaxes blood vessels and reduces blood pressure. It diffuses from endothelial cells into the underlying smooth muscle cells and induces vasodilation by stimulating NO-sensitive guanylyl cyclase. NO produced by the endothelial eNOS also diffuses into the blood and inhibits platelet aggregation and adhesion. In addition to these antihypertensive and antithrombotic actions, eNOS-derived NO also possesses multiple anti-atherosclerotic properties, including prevention of leukocyte adhesion to vascular endothelium and leukocyte migration into the vascular wall; inhibition of low-density lipoprotein (LDL) oxidation; and inhibition of vascular smooth muscle cell proliferation [3,4]. Genetic depletion of eNOS elevates blood pressure [5] and exacerbates diet-induced atherosclerosis in mouse models [6,7].

Recent studies suggest that eNOS-derived NO also promotes mitochondrial biogenesis [8], has antiobesogenic effects [9], and may be involved in the anti-aging effects and extension of lifespan induced by calorie restriction [10]. Depletion of the eNOS gene induces hyperinsulinemia and insulin resistance [11]. Overexpression of eNOS prevents weight gain in high fat-fed mice by stimulating mitochondrial biogenesis and activity in adipose tissues [9].

## 2. Resveratrol Targets

Resveratrol (3,5,4'-trihydroxy-trans-stilbene) is a polyphenol phytoalexin present in a variety of plant species, including *Veratrum grandiflorum* (white hellebore), *Polygonum cuspidatum* (Japanese knotweed), *Vitis vinifera* (grapes), *Arachis hypogaea* (peanuts) and *Morus rubra* (mulberries) [12,13,14]. The name “resveratrol” has been derived from its source; the compound is a resorcinol derivative from a *Veratrum* species.

Resveratrol is a molecule with many targets [15,16], which represents the molecular basis for its versatile pharmacological effects. The molecular targets of resveratrol include those that directly interact with resveratrol physically and others, which are modulated indirectly (e.g., through change at expression levels) [16]. Over 20 molecules have been identified that directly bind to resveratrol [16]. For its effects on endothelial NO, the following resveratrol targets are of particular importance: the NAD^+^-dependent, class III histone deacetylase sirtuin 1 (SIRT1), the AMP-activated protein kinase (AMPK), the nuclear factor-erythroid-derived 2-related factor-2 (Nrf2), and estrogen receptors (ER).

### 2.1. SIRT1

Resveratrol has been identified as a SIRT1 activator in an *in vitro* assay that uses a fluorogenic acetylated peptide derived from p53, a native SIRT1 substrate [17]. Dose-response experiments showed that resveratrol doubles the rate of SIRT1-mediated deacetylation at about 11 µM and a saturation of SIRT1 activation is reached at 100–200 µM [17].

The claim of resveratrol as a direct SIRT1 activator was questioned by subsequent studies, which showed that once the fluorophore was removed from the peptide substrate, the induction of SIRT1 activity by resveratrol was no longer detectable [18,19,20,21]. Later studies have revealed that the activation of SIRT1 by resveratrol depends on the structure of the SIRT1 substrate. SIRT1 can be activated by resveratrol *in vitro*, but only on certain peptide substrates [22]. Screening studies for substrate specificity indicate that hydrophobic side chains at +1 and +6 (relative to the acetylated lysine) are important. Natural SIRT1 substrates with a large hydrophobic residue (Trp, Tyr, or Phe) at position +1 (e.g., Forkhead box O factor FOXO3a) or positions +1 and +6 (e.g., proliferator-activated receptor-coactivator-1 α, PGC-1α) can be selectively activated by resveratrol [22]. It is plausible that the fluorophore tag attached to the substrates employed for the original SIRT1 activation screening mimic hydrophobic amino acids of natural substrates. This is likely to be the reason why SIRT1 can be activated by resveratrol on the fluorogenic substrates.

Thus, toward a subset of SIRT1 targets, resveratrol directly binds to and allosterically activates SIRT1 via a regulatory domain in the amino terminus [23]. The substrate-dependent effect of resveratrol on SIRT1 activity may explain why resveratrol shows SIRT1-dependent effects that overlap with, but are not identical to, the effects of SIRT1 overexpression and why resveratrol fails to stimulate SIRT1 activity against some substrates [23].

In addition, resveratrol may activate SIRT1 indirectly. Park *et al.* have recently shown that resveratrol, by inhibiting phosphodiesterase (PDE) enzymes, activates SIRT1 through a signaling cascade involving cAMP, Epac1, Ca^2+^, calcium/calmodulin-dependent kinase kinase β (CamKKβ) and AMPK [24]. AMPK activation improves NAD^+^ availability for SIRT1 by switching from carbohydrate to lipid as the main energy source [25] and by enhancing the expression of the NAD^+^-producing enzyme Nampt [25,26]. A second mechanism by which resveratrol indirectly activates SIRT1 involves the nuclear matrix protein lamin A, which has been identified as a protein activator of SIRT1 [27,28]. Lamin A activates SIRT1 by direct binding in the N terminus of SIRT1. Resveratrol enhances the binding of SIRT1 to lamin A and thus increases SIRT1 deacetylase activity [27]. Finally, the SIRT1-dependent effects of resveratrol *in vivo* may be partially mediated by resveratrol-induced upregulation of SIRT1 expression [8,29].

The activation of SIRT1 by resveratrol leads to changes in a broad range of biological processes, because SIRT1 itself is also a molecule with many targets. A number of SIRT1 target molecules have been identified, including histones, histone-modifying enzymes (e.g., the histone acetyltransferase p300), transcription factors and co-regulators, other cytosolic SIRT1 substrates (e.g., eNOS) and SIRT1 interacting proteins [30,31,32,33]. For instance, deacetylation of the RelA/p65 subunit of NF-κB by SIRT1 leads to NF-κB inhibition and anti-inflammatory effects [34]. By targeting FOXO transcription factors, p53, SREBP and PGC-1α, SIRT1 regulates the expression of diverse enzymes involved in cell cycle/apoptosis, stress defense, anti-aging processes, lipid metabolism and metabolic adaptation [23,30]. SIRT1 enhances eNOS activity by directly deacetylating eNOS at lysines 496 and 506 [35], and upregulates eNOS expression through FOXO factor-mediated mechanisms [29]. FOXO factors are also involved in the resveratrol-induced, SIRT1-mediated upregulation of antioxidant enzymes (see our recent review articles [36,37]).

### 2.2. AMPK

AMPK is not a direct target of resveratrol. AMPK can be phosphorylated and activated by LKB1 or CamKKβ. Resveratrol has been shown to activate LKB1 by preventing lipid peroxidation byproduct 4-hydroxy-2-nonenal [38] or by reducing ATP levels [39,40]. In contrast, activation of AMPK via increased intracellular Ca^2+^ is dependent on CamKKβ. Resveratrol has been shown to induce CamKKβ-mediated AMPK phosphorylation and activation by inhibiting PDE [24]. Whereas a high concentration (100–300 µM) of resveratrol is needed to activate AMPK by decreasing ATP, relatively low concentrations (<10 µM) is sufficient to activate AMPK by inhibiting PDE, without decreasing energy [24].

In addition, resveratrol has been shown to activate AMPK through SIRT1-mediated deacetylation and activation of LKB1 in hepatocytes and muscle cells [41,42]. Thus, there seems to be a cross-talk between AMPK and SIRT1. The two pathways can act synergistically to reinforce one another. AMPK may lead to the activation of SIRT1 by increasing NAD^+^ levels. On the other hand, SIRT1 can deacetylate and activate the AMPK upstream kinase LKB1, which, in turn, activates AMPK [43,44].

### 2.3. Nrf2

Nrf2 has also been identified as an (indirect) target of resveratrol [45]. Activation of Nrf2 by resveratrol leads to upregulation of several antioxidant enzymes (see our recent review articles [36,37]). Under basal conditions, Nrf2 interacts with Kelch-like ECH-associated protein 1 (Keap-1), a cytosolic repressor protein that limits Nrf2-mediated gene expression. Upon stimulation, Nrf2 is released from Keap-1 and translocates to the nucleus. It binds antioxidant-response element (ARE) and activates ARE-dependent transcription of phase II and antioxidant defense enzymes, such as heme oxygenase-1 (HO-1), NAD(P)H:quinoneoxidoreductase 1 (NQO1), and γ-glutamylcysteine synthetase (GCLC, the rate-limiting enzyme for glutathione synthesis) [45].

The molecular mechanism by which resveratrol activates Nrf2 is still unclear. Interestingly, significantly lower concentrations of resveratrol (<1 µM) are needed for the activation of Nrf2 than that for activating SIRT1 [45]. Such concentrations (that are sufficient for Nrf2 activation) can be achieved in the plasma when resveratrol is used as a dietary supplement [13].

### 2.4. Estrogen Receptors

As a polyphenolic phytoestrogen, resveratrol stimulates estrogen receptors (ER). Estrogens activate both classical genomic pathways regulating gene transcription via nuclear ER as well as non-genomic intracellular signaling pathways through membrane ER. Resveratrol binds ERα and ERβ with a *K_d_* in the micromolar range [46] and the EC_50_ for resveratrol activation of estrogen response element (ERE)-driven reporter gene activity has been shown to be 10 µM [46].

A subpopulation of ERα is associated with caveolae in the endothelial plasma membrane and coupled to eNOS in endothelial cells via a G protein [47]. Resveratrol has been shown to rapidly activate eNOS by stimulating the same non-genomic ER pathway as estrogens does [48,49]. Importantly, only nanomolar concentrations of resveratrol are need for this effect - concentrations that can be achieved in human blood after oral ingestion of red wine or grape juice.

In addition to this rapid effect on eNOS activity, ER has also been implicated in the upregulation of HO-1 (and possibly also in the downregulation of NADPH oxidases) by resveratrol [50].

## 3. Cardiovascular Effects of Resveratrol

Pre-clinical studies have demonstrated multiple beneficial effects of resveratrol in animal models of cardiovascular disease [36]. A number of clinical trials have been completed and some are ongoing.

### 3.1. Endothelial Progenitor Cells

Resveratrol improves endothelial progenitor cell (EPC) function [51,52,53] and delays EPC senescence [54,55]. SIRT1 [56,57] and eNOS [58] have been implicated in the effect of resveratrol on EPC. In a mouse model of hindlimb ischemia, exposure to red wine (containing 4–6 mg/L resveratrol) increases EPC number by 60% which is associated with increased capillary density [59]. EPC from preterm infants display an accelerated senescence and reduced SIRT1 levels. SIRT1 overexpression or resveratrol treatment reverse EPC senescence phenotype and rescue EPC dysfunction in a SIRT1-dependent manner [60].

### 3.2. Endothelial Function

The endothelium has emerged as the key regulator of vascular homeostasis. It has not merely a barrier function but also acts as an active signal transducer for circulating influences by production of a wide range of factors that regulate vascular tone, cellular adhesion, thrombogenesis, smooth muscle cell proliferation, and vessel wall inflammation [61]. Alteration in endothelial function precedes the development of morphological atherosclerotic changes and also contribute to lesion development and later clinical complications [61]. Endothelium-dependent vasodilation is most widely used for assessment of endothelial function.

Oral treatment with resveratrol improves endothelium-dependent vasodilation. This has been shown in diverse animal models of disease, such as hypertensive rats [62,63], hypertensive mice [63], diabetic rats [64], diabetic mice [65], and hypercholesterolemic rabbits [66]. In addition, improvement of endothelial function has also been observed in arteries from patients with hypertension and dyslipidemia *ex vivo* [67] and in overweight/obese men or post-menopausal women with untreated borderline hypertension *in vivo* [68].

### 3.3. Hypertension

Antihypertensive effects of resveratrol have been demonstrated in several animal models, including spontaneously hypertensive rats [63], ovariectomized, stroke-prone spontaneously hypertensive rats [62], angiotensin II-infused mice [63], a rat model of partial nephrectomy-induced cardiac hypertrophy [69], and in fructose-fed rats (an experimental model of insulin resistance) [70]. In overweight middle-aged men, 30 days of resveratrol treatment reduces blood pressure [71].

### 3.4. Atherosclerosis

Resveratrol improves lipid profiles in apolipoprotein E-knockout (ApoE-KO) mice [72] and in high-cholesterol diet-fed rats [73], although the effect in human is still uncertain [74]. Treatment with resveratrol reduces atherosclerosis in hypercholesterolemic rabbits [75] and in ApoE-KO mice [72,76].

### 3.5. Diabetes

Resveratrol has been shown to decrease blood glucose, protect pancreatic beta-cells from oxidative damage, and reduce diabetic vascular complications [77] as well as diabetic cardiomyopathy [78]. The anti-hyperglycemic effects of resveratrol have been demonstrated in streptozotocin (STZ)-induced (type 1) as well as in STZ-nicotinamide-induced (type 2) diabetes [79,80]. In mouse models of high fat-induced insulin resistance, resveratrol enhances insulin sensitivity [81,82]. The metabolic effects of resveratrol require both SIRT1 [81] and AMPK [82]. In a randomized double-blind crossover study, treatment of 11 healthy, obese men with 150 mg/day resveratrol for 30 days induces modest metabolic changes mimicking the effects of calorie restriction [71].

## 4. Effects of Resveratrol on Endothelial Nitric Oxide 

**Figure 1:**
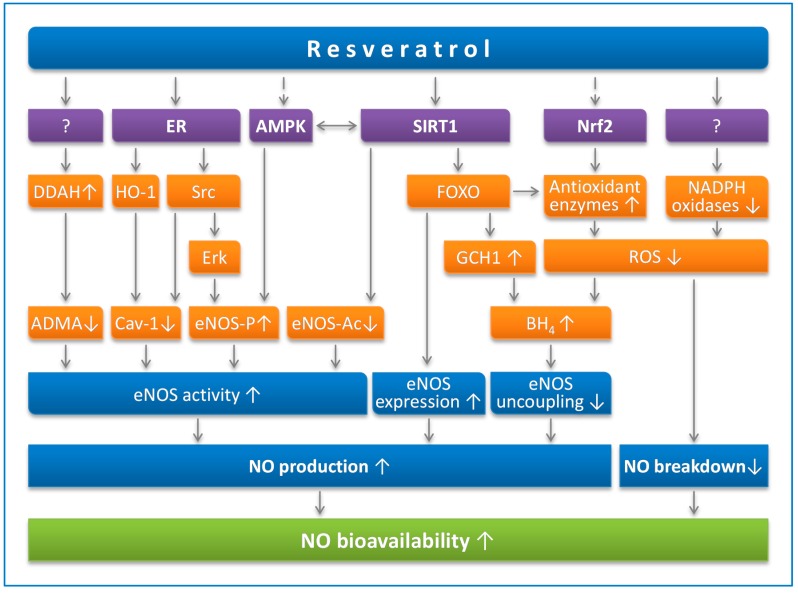
Resveratrol enhances NO production and prevents NO breakdown. Resveratrol can activate sirtuin 1 (SIRT1) directly (in a substrate-dependent manner) or indirectly (by either inhibiting phosphodiesterases or enhancing the effect of lamin A). SIRT1 stimulates endothelial NO synthase (eNOS) activity through deacetylation, enhances eNOS expression by deacetylating Forkhead box O (FOXO) transcription factors and prevents eNOS uncoupling by upregulating GTP cyclohydrolase 1 (GCH1), the rate-limiting enzyme in tetrahydrobiopterin (BH_4_) biosynthesis. AMP-activated protein kinase (AMPK) and nuclear factor-erythroid-derived 2-related factor-2 (Nrf2) are indirect targets of resveratrol. AMPK phosphorylates eNOS at serine 1177. eNOS can also be phosphorylated by Erk1/2, which is stimulated by a pathway involving estrogen receptors (ER) and the tyrosine kinase Src. Caveolin-1 (Cav-1) is an eNOS-interacting protein that negatively regulates eNOS activity. Asymmetric dimethylarginine (ADMA) is an endogenous eNOS inhibitor that is degraded by dimethylarginine dimethylaminohydrolase (DDAH). The resveratrol targets for DDAH upregulation or for NADPH oxidase downregulation have not been identified so far.

At least part of the cardiovascular effects of resveratrol are mediated by endothelial NO. Indeed, resveratrol enhances NO production through multiple mechanisms and prevents NO breakdown by reducing oxidative stress (Figure 1).

### 4.1. Resveratrol Prevents eNOS Uncoupling 

Under physiological conditions, eNOS produces NO, which represents a key element in the vasoprotective function of the endothelium [1,3,4]. Under pathological conditions associated with oxidative stress, however, eNOS may become dysfunctional. Oxidative stress contributes markedly to endothelial dysfunction, primarily due to rapid oxidative inactivation of NO by excess superoxide. In addition, the persisting oxidative stress renders eNOS uncoupled (*i.e.*, uncoupling of O_2_ reduction from NO synthesis), such that it no longer produces NO, but superoxide [83,84,85].

Numerous mechanisms have been proposed to play a role in eNOS uncoupling [1,4]. Among these, depletion of BH_4_, an essential cofactor for the eNOS enzyme, is likely to be a major cause for eNOS uncoupling and endothelial dysfunction. Superoxide can modestly and peroxynitrite strongly oxidize BH_4_, leading to BH_4_ deficiency [86]. Another important cause of eNOS uncoupling is a deficiency of l-arginine due to upregulation of arginase expression/activity [87].

Uncoupling of eNOS is a crucial mechanism contributing significantly to endothelial dysfunction and cardiovascular disease. It not only reduces NO production, but also potentiates the pre-existing oxidative stress. The overproduction of reactive oxygen species (ROS; e.g., superoxide and subsequently peroxynitrite) by uncoupled eNOS in turn enhances oxidation of BH_4_ and upregulation of arginase expression/activity [88], creating a vicious circle.

We have recently shown that resveratrol prevents eNOS uncoupling in cardiovascular tissues. As mentioned above, BH_4_ deficiency is a major cause of eNOS uncoupling under pathological conditions. BH_4_ supplementation is capable of correcting eNOS dysfunction in several types of pathophysiology [4,83]. The ApoE-KO mice are a mouse model of atherosclerosis. These animals show enhanced ROS production and increased oxidative degradation of BH_4_ in the aorta [89,90] and heart [91]. Both the aortic and cardiac superoxide production can be reduced by the NOS inhibitor l-NAME, indicating that eNOS contributes to superoxide production in this pathological model, *i.e.*, eNOS is in an uncoupled state.

Treatment of ApoE-KO mice with resveratrol leads to a marked reduction of cardiac superoxide production. Moreover, the superoxide level in resveratrol-treated animals cannot be lowered any further by l-NAME [91]. These data suggest that eNOS is no longer producing superoxide in resveratrol-treated ApoE-KO mice, *i.e.*, eNOS uncoupling is reversed by resveratrol.

The tissue levels of BH_4_ depend on its biosynthesis and its degradation/oxidation [92]. The reversal of eNOS uncoupling by resveratrol is likely to be attributable to both mechanisms, *i.e.*, stimulation of BH_4_ biosynthesis and prevention of BH_4_ oxidation. BH_4_ is synthesized from GTP via a *de novo* pathway with GTP cyclohydrolase 1 (GCH1) acting as the rate-limiting enzyme. Resveratrol treatment enhances the expression of GCH1 (SIRT1-dependently) and BH_4_ biosynthesis [91]. In addition, resveratrol decreases the cardiac content of superoxide and peroxynitrite, and thereby decreases BH_4_ oxidation [91].

### 4.2. Resveratrol Enhances eNOS Expression 

Our previous studies have demonstrated that treatment of cultured human endothelial cells with resveratrol [93] or red wines rich in resveratrol [94,95] enhances the mRNA and protein expression of eNOS. Resveratrol increases the activity of eNOS promoter as well as eNOS mRNA stability, indicating both transcriptional and posttranscriptional mechanisms. Experiments with estrogen receptor antagonists ICI 182780 and RU 58668 indicate that effect of resveratrol on eNOS expression is independent of estrogen receptors [93].

SIRT1 is implicated in the effect of resveratrol on eNOS expression. An endothelium-specific overexpression of SIRT1 leads to an enhanced eNOS expression in mice [96]. In human coronary arterial endothelial cells, resveratrol-induced eNOS expression can be prevented by the knockdown of SIRT1 [8]. Our recent data indicate that FOXO factors are likely to be the downstream SIRT1 targets for this effect of resveratrol [29].

The predominant FOXO isoforms in human endothelial cells are FOXO1 and FOXO3a [97]. Resveratrol enhances the expression and DNA-binding activity of FOXO factors [29]. A previous study showed that FOXO factors are negative regulators of eNOS expression. Knockdown of FOXO1 or FOXO3a led to an upregulation of eNOS expression [97]. We also observed an upregulation of eNOS by FOXO1 siRNA [29]. Even FOXO factors are likely to be eNOS suppressors under basal conditions, under resveratrol-stimulated conditions, FOXO factors seem to be positive regulators of eNOS transcription. Resveratrol-induced eNOS expression can be completely blocked by a combined knockdown of FOXO1 and FOXO3a [29]. Knockdown of only one FOXO factor, on the other hand, was not sufficient to prevent the effect of resveratrol [29]. It is known that FOXO factors have both distinct and overlapping functions in organisms. Therefore, it is conceivable that the role of one member can be taken over by the other.

### 4.3. Resveratrol Stimulates eNOS Activity 

In addition to its effect on eNOS expression (within hours), resveratrol also increases enzymatic activity of eNOS acutely (within minutes) [29,93].

The enzymatic activity of eNOS is increased in response to shear stress and numerous agonists. This activation is mediated by different cellular events such as increase of intracellular Ca^2+^, interaction with substrate, co-factors, adaptors and regulatory proteins, and through shuttling between distinct subcellular domains [2]. In addition, eNOS activity is also regulated by post-translational modification of the eNOS protein (e.g., phosphorylation and acetylation) and by methylarginines.

#### 4.3.1. eNOS Phosphorylation

eNOS is phosphorylated on diverse serine, threonine and tyrosine residues [2]. The best studied phosphorylation site is serine 1177 of human eNOS, which is associated with enhanced eNOS activity [2].

Treatment of endothelial cells with nanomolar concentrations of resveratrol leads to the rapid phosphorylation of eNOS at serine 1177 and an increase in eNOS enzymatic activity [48,49]. This effect of resveratrol has been shown to be mediated by estrogen receptors. Whereas both estrogen receptors ERα and ERβ may play a role in bovine aortic endothelial cells [49], in human umbilical vein endothelial cells, however, the activation of eNOS by resveratrol is likely to be mediated by ERα that is localized in a “signalsome complex” within caveolae [48]. Resveratrol activates ERα, leading to eNOS activation via a signal cascade that involves the G-protein Gα, Caveolin-1 (Cav-1), the tyrosine kinase c-Src and the MAP kinase Erk1/2 (Figure 1).

The therapeutic relevance of ERα-mediated eNOS activation by resveratrol has been demonstrated in a mouse model of restenosis [98]. In a NO-dependent manner, resveratrol markedly reduces the endothelial denudation-induced neointimal hyperplasia of the carotid artery. Both effects (improvement of NO production and inhibition of neointima formation) of resveratrol are lost in ER-α knockout mice [98].

AMPK can also phosphorylate eNOS at serine 1177 [2]. In cultured human umbilical vein endothelial cells, resveratrol at concentrations of 10–100 µM has been shown to activate AMPK, which is associated with eNOS phosphorylation at serine1177 and enhanced NO production [99]. In mouse aortic rings, resveratrol induces endothelium-dependent vasodilatation and alleviates high glucose-mediated endothelial dysfunction [99]. The abovementioned effects of resveratrol can be prevented by pharmacological antagonism of AMPK, indicating the involvement of this kinase [99]. AMPK-mediated eNOS phosphorylation by resveratrol has also been observed in superior thyroid arteries from patients with hypertension and dyslipidemia [67].

The resveratrol-induced eNOS phosphorylation and activation may be of therapeutic importance. The concentration of resveratrol required for the ER-mediated effects can be achieved in human circulation after modest wine consumption [13]. Thus, the resveratrol-induced eNOS phosphorylation and activation may be implicated in the protective effects of red wine. In diabetic mice, resveratrol restores endothelial function by inhibiting TNFα-induced activation of NADPH oxidase and preserving eNOS phosphorylation at serine 1177 [65].

#### 4.3.2. eNOS Acetylation

The enzymatic activity of eNOS is also regulated by acetylation. Short-term treatments of endothelial cells with resveratrol lead to eNOS deacetylation at lysines 496 and 506 in the calmodulin-binding domain, which is associated with an increase in eNOS activity. This effect is mediated by SIRT1, as demonstrated by siRNA experiments. Furthermore, SIRT1 and eNOS colocalize and coprecipitate in endothelial cells, indicating direct interaction between SIRT1 and eNOS [35]. Calorie restriction of mice leads to deacetylation of eNOS suggesting that SIRT1-mediated eNOS deacetylation may represent part of the mechanisms underlying the improvement of endothelial function in response to calorie restriction [35].

Oxidative stress downregulates SIRT1, leading to acetylation of eNOS and reduced NO production in endothelial cells [100]. Pre-treatment of endothelial cells with resveratrol significantly attenuates the effect of oxidative stress on SIRT1 levels and on eNOS acetylation [100].

Interestingly, low-dose aspirin has been shown to increase eNOS activity by acetylating eNOS in endothelial cells [101]. The activation of eNOS by aspirin is attributable to an enhanced binding of eNOS to calmodulin promoted by acetylation of lysine 609 in the autoinhibitory domain of bovine eNOS (corresponding to position 607 in human eNOS). The aspirin-induced eNOS acetylation at lysine 607 can be reversed by histone deacetylase 3 (HDAC3), but not by SIRT1 [102]. High-dose of aspirin induces acetylation on serines 765 and 771 of human eNOS (767 and 773 in bovine eNOS) in platelets [103]. Also this effect is unrelated to SIRT1 or resveratrol.

#### 4.3.3. Endogenous eNOS Inhibitors

Methylarginines, such as monomethyl-arginine (l-NMMA), asymmetric dimethylarginine (ADMA) and symmetric dimethylarginine (SDMA), are endogenously released when proteins containing methylated arginine residues are degraded [104]. Elevated plasma levels of ADMA have been associated with cardiovascular events and mortality. ADMA has been identified as an endogenous eNOS inhibitor, although NO-independent effects of ADMA have also been reported [105]. ADMA is degraded by the intracellular enzyme dimethylarginine dimethylaminohydrolase (DDAH). Decreased DDAH expression/activity is evident in disease states associated with endothelial dysfunction [106].

Treatment of bovine aortic endothelial cells with high concentration of glucose leads to a reduction of DDAH expression and activity, which is associated with accumulation of intracellular ADMA. Pre-treatment with resveratrol (or piceatannol) restores DDAH activity and normalizes ADMA level in a dose-dependent manner [107]. Similarly, in a glucose-induced endothelial cell senescence model, the decreased DDAH activity and increased ADMA levels can be normalized by BTM-0512, a derivative of resveratrol [108]. The beneficial effects of BTM-0512 on high glucose-induced senescence can be blocked by splimtomicin, an inhibitor of SIRT1, or by silencing DDAH2 expression, indicating an involvement of SIRT1 in the DDAH/ADMA pathway [108].

#### 4.3.4. Cav-1

Cav-1 is one of the proteins that interact with eNOS in eNOS signalosome [2]. Cav-1 negatively regulates eNOS activity [109]. Cav-1 deficiency is associated with reduced atherosclerosis in ApoE-KO mice [110].

In a rat myocardial infarction model, the high cholesterol diet-induced complications such as increased lipid levels, Cav-1 expression and Cav-1/eNOS association, as well as reductions in myocardial functions, can be normalized with resveratrol therapy [73]. The downregulation of Cav1- is likely to be mediated by HO-1; HO-1 overexpression decreases Cav-1 expression [73]. Similar effects have been observed in the rat model of STZ-induced diabetes. The increased Cav-1 expression and Cav-1/eNOS association in diabetic myocardium is reduced by resveratrol treatment [111].

Collectively, the effect of resveratrol on Cav-1 occurs at two different levels: downregulation of Cav-1 expression (which needs hours or days) and inhibition of Cav-1/eNOS association (which happens within minutes). Both effects have been observed in the heart [73,111] as well as in endothelial cells [48,112]. The acute reduction Cav-1/eNOS association (and thus enhance eNOS activity) is likely to be due to Src kinase-mediated Cav-1 phosphorylation on Tyr-14 [48]. This effect of resveratrol is dependent on estrogen receptors [48].

### 4.4. Resveratrol Reduces NO Breakdown 

The bioavailability of NO depends on one hand on NO production and on the other hand on NO breakdown by superoxide. Antioxidative effects of resveratrol represent another mechanism improving NO bioavailability. By reducing ROS levels, resveratrol not only prevent eNOS uncoupling (see above), but also decreases superoxide-mediated NO inactivation.

As a polyphenolic compound, resveratrol has been shown to be a scavenger of hydroxyl, superoxide, metal-induced radicals [113,114], and H_2_O_2_ [115]. However, the direct antioxidant effects of resveratrol are rather poor; resveratrol is less potent than other well established antioxidants such as ascorbate and cysteine [116]. Thus, the protective effects of resveratrol against oxidative injury are more likely to be attributed to the upregulation of the endogenous cellular antioxidant systems rather than its direct ROS scavenging activity [36,37].

Resveratrol prevents superoxide production from uncoupled eNOS [91], from NADPH oxidases [91,117] and from mitochondria [8,118].

In addition, resveratrol accelerates ROS detoxification by upregulating antioxidant enzymes in cardiovascular tissues, including all three SOD isoforms [91,119], GPx1 [91,119], catalase [91,120], NQO1 [45], HO-1 [45], and GCLC [45]. The upregulation of SOD1 [91], SOD2 [91,118], GPx1 [91] and catalase [120] by resveratrol has been shown to be (partially) mediated by SIRT1, in some cases involving FOXO1 [121] or FOXO3a [30,122] as downstream SIRT1 target. Nrf2-dependent transcriptional activation is implicated in resveratrol-induced expression of NQO1 and HO-1, GCLC, GPx1 [45,123], and SOD2 [67].

## 5. Conclusions 

Resveratrol improves endothelial function, exerts antihypertensive, anti-atherosclerotic, anti-hyperglycemic effects, and enhances insulin sensitivity. Part of the protective effects of resveratrol are attributable to eNOS-derived NO. Resveratrol enhances NO bioavailability by stimulating NO production and by preventing superoxide-mediated NO breakdown. Multiple pathways are activated by resveratrol and this represents the molecular basis for the versatile pharmacological effects of this compound.
